# ICU mortality following ICU-acquired primary bloodstream infections according to the type of pathogen: A prospective cohort study in 937 Germany ICUs (2006-2015)

**DOI:** 10.1371/journal.pone.0194210

**Published:** 2018-03-08

**Authors:** Frank Schwab, Christine Geffers, Michael Behnke, Petra Gastmeier

**Affiliations:** 1 Charité –Universitätsmedizin Berlin, corporate member of Freie Universität Berlin, Humboldt-Universität zu Berlin, and Berlin Institute of Health, Institute for Hygiene and Environmental Medicine, Berlin, Germany; 2 National Reference Centre for Surveillance of Nosocomial Infections, Berlin, Germany; Universitatsklinikum Hamburg-Eppendorf, GERMANY

## Abstract

**Objective:**

Mortality due to intensive care unit (ICU) acquired primary blood stream infections (PBSI) is related primarily to patient co-morbidities, types of pathogens and quality of care. The objective of this study is to determine the impact of various types of pathogen on ICU mortality.

**Methods:**

Data from the German National Nosocomial Infection Surveillance System of patients with PBSI from 2006 to 2015 was used for this analysis. A BSI is primary when the pathogen recognized is not related to an infection on another site. Only mono-microbial infections stratified into the 13 pathogens most frequently causing PBSI were considered. Univariate and multivariate risk factor analyses were performed using the following risk factors: Sex, age, length of stay, device use, time until onset of PBSI, type and size of hospital, type of ICU and type of pathogen. ICU mortality following *S*.*aureus* PBSI was used as the reference value.

**Results:**

A total of 4,556,360 patients with 16,978,882 patient days from 937 ICUs were considered in the analysis. Of 14,626 PBSI in total, 12,745 mono-microbial PBSI were included. The ICU mortality was 18.6%. Compared with *S*.*aureus* and adjusted by age, sex and type of ICU, *S*.*maltophilia* was associated with significantly higher ICU mortality (OR 1.71; 95%CI 1.19–2.47) as followed by Enterococci (OR 1.20; 95%CI 1.06–1.36), *E*.*coli* (OR 1.24; 95%CI 1.02–1.49), *C*.*albicans* (OR 1.37; 95%CI 1.16–1.61), non albicans Candida *spp*. (OR 1.49; 95%CI 1.18–1.88) and *P*.*aeruginosa* (OR 1.49; 95%CI 1.21–1.84). Coagulase negative Staphylococci were associated with significant lower ICU mortality (OR 0.86; 95%CI 0.75–0.99).

**Conclusion:**

Because of the limitation of the study in adjusting for severity of illness and appropriateness of therapy, the differences between the pathogens may not only be explained by differences in virulence, but may reflect the prognosis after receiving the microbiological results and may therefore be useful for intensive care physicians.

## Introduction

Primary bloodstream infections (PBSI) in intensive care units (ICU) are associated with high mortality. This mortality is related to patient factors (sex, age and underlying diseases), adequate antibiotic therapy ICU factors (staff to patient ratios, safety climate, etc.) and the pathogens responsible. Some pathogens (e.g. *P*.*aeruginosa*) and some multi-drug resistant variants of pathogens (e.g. multiresistant *A*.*baumannii*) are known to be associated with significantly higher ICU mortality [1–3]. However, such information is not available for many other pathogens that frequently cause primary bloodstream infections (PBSI).

The reason for this lack of information is the need to analyze large numbers of PBSI cases. In addition, it would be preferable to diagnose infections by applying definitions designed for surveillance and not those derived from other databases, e.g. discharge data. Furthermore, information about the most important risk factors needs to be available, including the time interval between ICU admission and the onset of infection. Therefore, only a few studies have been able to provide comparable information about mortality based on the type of pathogen [3–8]. Many included only a small number of species or did not consider information for such frequently occurring pathogens like *E*.*coli* or Enterococci.

The ICU component of the German National Nosocomial Infection Surveillance System (KISS) includes a large number of ICUs [9]. In the case of healthcare-associated PBSI, it records not only the type of pathogen isolated from relevant microbiological samples, but also the time from admission to the ICU until occurrence of PBSI. This database allows the analysis of differences between various pathogens that cause PBSI in ICUs in ICU mortality.

The objective of this study is to determine the impact of the type of pathogen on ICU mortality following a ICU-acquired PBSI.

## Material and methods

### Ethics statement

All data was anonymous and was collected in accordance with the German recommendations for good epidemiological practice with respect to data protection ((DAE) AEMdDAE (2013) Leitlinien und Empfehlungen zur Sicherung von Guter Epidemiologischer Praxis (GEP): http://www.gesundheitsforschung-bmbf.de/_media/GEP.pdf. Accessed 05 May 2017). A federal law, the German Protection against Infection Act (Infektionsschutzgesetz §23) regulates the prevention and management of infectious disease in humans. All hospitals are obliged to continuously collect and analyse healthcare-associated infections and drug-resistant pathogens (German Protection against Infection Act. "Gesetz zur Verhütung und Bekämpfung von Infektionskrankheiten beim Menschen" (2013): http://www.gesetze-im-internet.de/ifsg/index.html. Accessed 05 May 2017). This data is reported regularly to the National Reference Centre for the Surveillance for Nosocomial Infections. Ethical approval and informed consent were, therefore, not required.

### Data collection

Surveillance data from a ten-year period from January 2006 until December 2015 was used for the analysis. ICU-KISS is a unit-based surveillance system like the ICU component of the American National Healthcare Safety Network (NHSN). During this period, CDC definitions were used to diagnose PBSI [10]. A primary BSI was defined as a positive blood culture with one (or more) pathogen(s) which was not related to an infection on another site. For a BSI with skin germs, for example with coagulase negative Staphylococci (CNS), the definition also requires further clinical signs and the physician starts according to an antibiotic therapy. However, beginning in 2011 a new CDC definition of PBSI with skin germs was introduced [11]. Since that time two separate blood cultures are required for skin germs in combination with other clinical signs. The PBSI was defined as CVC-associated when the patient that had a CVC at the time of or within 48 hours before onset of the infection.

In the case of an ICU-acquired PBSI, the surveillance staff has to record the time to BSI, the ICU mortality and the pathogens identified in the blood culture. Up to four pathogens can be recorded per infection. Only mono-microbial infections were considered in the analysis. The 13 most common organisms causing PBSI were analyzed individually: coagulase negative Staphylococci (CNS), Enterococcus *spp*., *S*.*aureus*, *C*.*albicans*, non-albicans-Candida *spp*., *E*.*coli*, Klebsiella *spp*., Enterobacter *spp*., *P*.*aeruginosa*, Serratia *spp*., Acinetobacter *spp*., Proteus *spp*., and *S*.*maltophilia*. All other pathogens were analyzed as type “other”. In addition, for the 13 organisms specified we categorized the organisms into the pathogen groups (gram-positive/gram-negative bacteria/fungi) and multi-drug resistance groups (non-MDR/MDR). MDR was defined as Methicillin resistant for *S*.*aureus*, Vancomycin resistant for *E*.*faecalis* and *E*.*faecium*, and a resistance to at least three antibiotic groups for *P*.*aeruginosa*, Acinetobacter *spp*., Enterobacter *spp*., *E*.*coli*, *S*.*maltophilia*, Klebsiella *spp*. and/or the resistance mechanisms ESBL for *K*.*pneumonia* and *E*.*coli*.

### Statistical analysis

In our analysis, the primary outcome was ICU mortality following ICU-acquired PBSI. Univariable and multivariable risk factor analyses were performed to identify organisms *(pathogen groups and/or multidrug resistance groups)* associated with increased or decreased risk of ICU mortality as well as additional related factors and confounders, such as sex (male/female), age group (≤45/46-55/56-65/66-75/>75years), time from admission to the ICU until onset of PBSI(days), CVC associated with PBSI (no/yes), type of ICU (8 categories), size of ICU (</≥12 beds), type of hospital (tertiary care yes/no), and size of hospital (</≥600 beds), season(Dec-Feb/Mar-May/Jun-Aug/Sep-Nov) and long-term time trends (year). In the univariable analysis, differences were tested by Chi-Square test or Wilcoxon rank-sum test. Parameters with more than two categories were tested as binary parameters.

In the multivariable analysis, a logistic regression analysis was performed. Since observations within an ICU are not statistically independent due to varying patient populations (especially the treatment management), adjusted odds ratios (OR) with 95% confidence intervals (CI) were calculated. They were based on generalized estimating equation (GEE) models which account for this clustering effect by using an exchangeable correlation structure. The multivariable model building strategy was performed stepwise backward, from a full model with all parameters, non-significant parameters were excluded with the significance level of p = 0.05 for excluding a parameter from the model in the type III test. *S*.*aureus* was used as reference micro-organisms because this pathogen frequently causes PBSI, and it is very likely that, when detected in a blood culture, it is the responsible pathogen, in contrast to coagulase negative Staphylococci, which are often regarded as skin contaminants. In addition, experts agree that *S*.*aureus* is a common cause of severe bloodstream infections, and mortality and morbidity of *S*.*aureus* bacteremia still remain considerably high [12–13]. Furthermore, other studies have provided substantial information about *S*.*aureus* mortality which can be used for comparison [8], [14]. For epidemiological reasons, age group and sex were included in all models. P-values less than 0.05 were considered significant.

Beside age, gender and time to PBSI, no more individual patient-based parameters are available in the multivariable analysis which describe the severity of illness of the patients. However, structure parameters, such as type and size of ICU, and type and size of the hospital where the ICU is located, were considered in the multivariable analysis.

In addition, to better characterize risk potential of patients, we conducted a further multivariable analysis which considered the monthly mean of length of stay (LOS), device use (central venous catheter, invasive ventilation and urinary tract catheter) and bed occupation of the ICU in which the PBSI occurs. These results do not differ from the results without these parameters. Therefore, we present the results without these parameters.

All analyses were performed using SPSS [IBM SPSS statistics, version 23, Somer, NY, USA] and SAS 9.4 [SAS Institute, Cary, NC, USA].

## Results

A total of 4,556,360 patients with 16,978,882 patient days from 937 ICUs (> = 1 PBSI) were considered in the analysis. Table 1 describes the characteristics of the ICUs included.

**Table 1:** Characteristics of 973 intensive care units (ICU) included in the study, ICU-KISS, 2006–2015.

Parameter	Description	Number	Percentage
ICUs	total	937	100.0%
Surveillance time of ICU	Months (median, IQR)	56	30–95
Size of ICU	ICU beds (median, IQR)	12	8–15
Size of hospital	Hospital beds (median, IQR)	439	270–749
Type of hospital	Non tertiary care	659	70.4
	Tertiary care	278	29.6
Type of ICU	Interdisciplinary	495	52.8%
	Medical	140	14.9%
	Surgical	162	17.3%
	Neurosurgical	24	2.6%
	Cardiosurgical	26	2.8%
	Neurological	19	2.0%
	Pediatric	26	2.8%
	Other ^a^	45	4.8%
Length of stay	Days (median, IQR)	3.7	2.9–5.3
CVC utilization rate	CVC days per 100 pd (median, IQR)	65.9	51.0–79.8
Ventilator utilization rate	Ventilator days per 100 pd (median, IQR)	35.5	25.4–49.5
UC utilization rate	UC days per 100 pd (median, IQR)	84.0	72.8–91.7
Incidence density PBSI	PBSI/1000 pd (median, IQR)	0.64	0.33–1.14

PBSI, primary bloodstream infection; CVC, central venous catheter; UC, urinary catheter; pd, patient days; IQR, interquartile range.

^a^ other than Medical-surgical, Surgical, Medical, Cardiothoracic, Neurosurgical, Neurological or Pediatric.

A total of 14,626 PBSI were recorded, that is, an incidence of 0.32 per 100 patients or 0.86 per 1000 patient days. 95% of the PBSI were CVC-associated and the proportion does not change during the study period. For 12,745 PBSI, only one pathogen was isolated from the blood culture (87.1%).

The overall ICU mortality was 18.6% for PBSI. Mortality in multi-microbial PBSI was 24.1% (RR = 1.29, 95%CI 1.17–1.43, p<0.001 compared to mono-microbial PBSI).

Time in the ICU until onset of PBSI was in median 14 days and differs depending on the type of pathogen, PBSI were observed early in the case of *E*.*coli* (11 days) or *S*.*aureus* (12 days), but late if Klebsiella *spp*., non-albicans Candida *spp*. (both 16 days), *P*.*aeruginosa* (18 days) or *S*.*maltophilia* (19 days) were found.

Table 2 shows mortality following mono-microbial PBSI for the various risk groups, confounders and pathogens in ICU-KISS from 2006 to 2015. Significances were found for sex, age, type of ICU and hospital also for type of organisms, organism groups and multidrug resistance groups responsible.

**Table 2:** Results of univariate analysis for mortality in intensive care units (ICU) following mono-microbial primary bloodstream infections (PBSI) according to the type of organism and further risk factors and confounders, ICU-KISS, 2006–2015.

Parameter	Category	No. not died	No. died	Mortality (%)	p-value^a^
Patients with BSI	Total	10369	2376	18.6	
Type of organism	CNS	3579	641	15.2	<0.001
	Enterococcus *spp*.	1636	471	22.4	<0.001
	*S*. *aureus*	1522	338	18.2	0.573
	*Candida albicans*	510	166	24.6	<0.001
	*E*. *coli*	434	125	22.4	0.021
	Klebsiella *spp*.	498	102	17.0	0.290
	Enterobacter *spp*.	366	68	15.7	0.105
	*P*. *aeruginosa*	242	84	25.8	0.001
	non-albicans-Candida *spp*.	172	64	27.1	0.001
	Serratia *spp*.	162	27	14.3	0.121
	Acinetobacter *spp*.	91	21	18.8	0.977
	Proteus *spp*.	72	12	14.3	0.304
	*S*. *maltophilia*	48	19	28.4	0.041
	Other organisms ^b^	1037	238	18.7	0.981
Time to onset of infection	Days (median)	14	14		0.958
Gender	Male	6989	1541	18.1	0.017
Age	Years (median)	68	73		<0.001
Age group	≤45 years	2563	273	9.6	<0.001
	46–55 years	1990	399	16.7	0.007
	56–65 years	3010	730	19.5	0.102
	>65 years	2806	974	25.8	<0.001
CVC associated ^g^	Yes	9846	2276	18.8	0.089
Size of hospital	≥600 beds	5372	1224	18.6	0.796
Size of ICU	≥12 beds	7264	1637	18.4	0.267
Type of hospital	Tertiary care	4778	968	16.8	<0.001
Type of ICU	Interdisciplinary	4668	1098	19.0	0.292
	Medical	1437	417	22.5	<0.001
	Surgical	2482	525	17.5	0.057
	Neurosurgical	360	26	6.7	<0.001
	Cardiosurgical	437	157	26.4	<0.001
	Neurological	215	23	9.7	<0.001
	Pediatric	324	19	5.5	<0.001
	Other ^h^	446	111	19.9	0.426
Year of BSI	2006	687	157	18.6	0.426
	2007	784	159	16.9	0.975
	2008	830	191	18.7	0.956
	2009	958	197	17.1	0.147
	2010	1000	230	18.7	0.957
	2011	1051	225	17.6	0.329
	2012	1120	251	18.3	0.736
	2013	1230	299	19.6	0.329
	2014	1335	318	19.2	0.505
	2015	1374	349	20.3	0.065
Season	Winter (Dec-Feb)	2396	577	19.4	0.221
	Spring (Mar-May)	2613	620	19.2	0.366
	Summer (Jun-Aug)	2797	609	17.9	0.182
	Autumn (Sep-Nov)	2563	570	18.2	0.457
Pathogen groups	Gram-positive ^d^	6737	1450	17.7	<0.001
(N = 11470)	Gram-negative ^e^	1913	458	19.3	0.342
	Fungi ^f^	682	230	25.2	<0.001
Multi-drug (MDR) resistance groups (N = 6065)	MDR ^c^	779	265	25.4	<0.001

No., number; CNS, Coagulase negative staphylococci; CVC, central venous catheter; pd, patient days.

^a^ p-values, Chi-square test or Wilcoxon rank sum test

^b^ other than the 13 most common organisms causing PBSI: coagulase negative Staphylococci (CNS), Enterococcus *spp*., *S*. *aureus*, *C*.*albicans*, non-albicans-Candida *spp*., *E*. *coli*, Klebsiella *spp*., Enterobacter *spp*., *P*. *aeruginosa*, Serratia *spp*., Acinetobacter *spp*., Proteus *spp*., and *S*. *maltophilia;*

^c^ MDR, Multi-drug resistance includes Methicillin resistant *S*.*aureus*, Vancomycin resistant *E*.*faecalis* and E.*faecium*, and multidrug resistance defined as a resistance to at least 3 antibiotic groups for *P*.*aeruginosa*, Acinetobacter *spp*., Enterobacter *spp*., *E*.*coli*, *S*.*maltophilia*, Klebsiella *spp*. and/or the resistance mechanisms ESBL for *Klebsiella pneumonia* and *E*.*coli*, MDR compared to Non-MDR, includes Vancomycin sensible Enterococcus *spp*., Methicillin sensible *S*. *aureus* and non-multi-drug resistance for *E*. *coli*, Klebsiella *spp*., Enterobacter *spp*., *P*. *aeruginosa*, Acinetobacter *spp*., *S*. *maltophilia*

^d^ includes coagulase negative Staphylococci (CNS), Enterococcus *spp*., *S*. *aureus;*

^e^ includes *E*. *coli*, Klebsiella *spp*., Enterobacter *spp*., *P*. *aeruginosa*, Serratia *spp*., Acinetobacter *spp*., Proteus *spp*., and *S*. *maltophilia;*

^f^ *C*. *albicans*, non-albicans-Candida spp.

^g^ device present <48h before diagnosis of infection

^h^ other than Medical-surgical, Surgical, Medical, Cardiothoracic, Neurosurgical, Neurological or Pediatric.

**Fig 1:**
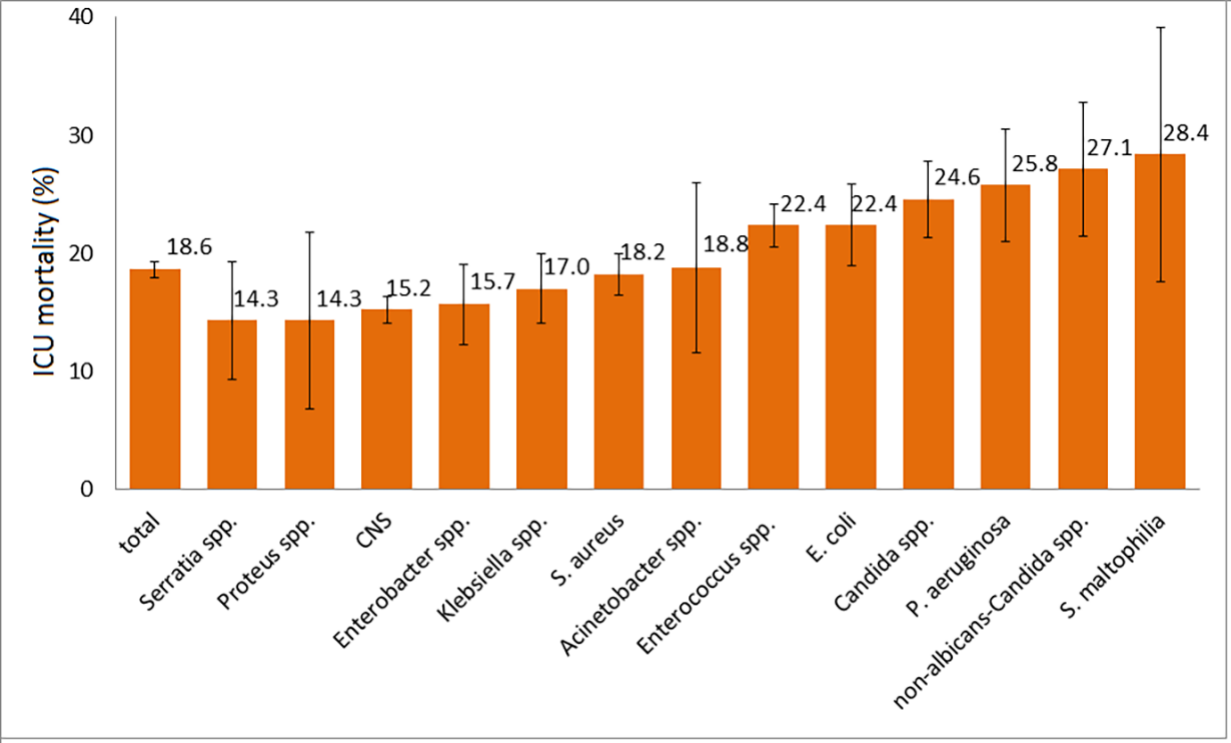
ICU mortality in patients with ICU-acquired primary bloodstream infections according to the type of pathogen. ICU, intensive care unit; CNS, coagulase negative staphylococci; Whiskers represent 95% confidence interval.

**Fig 2:**
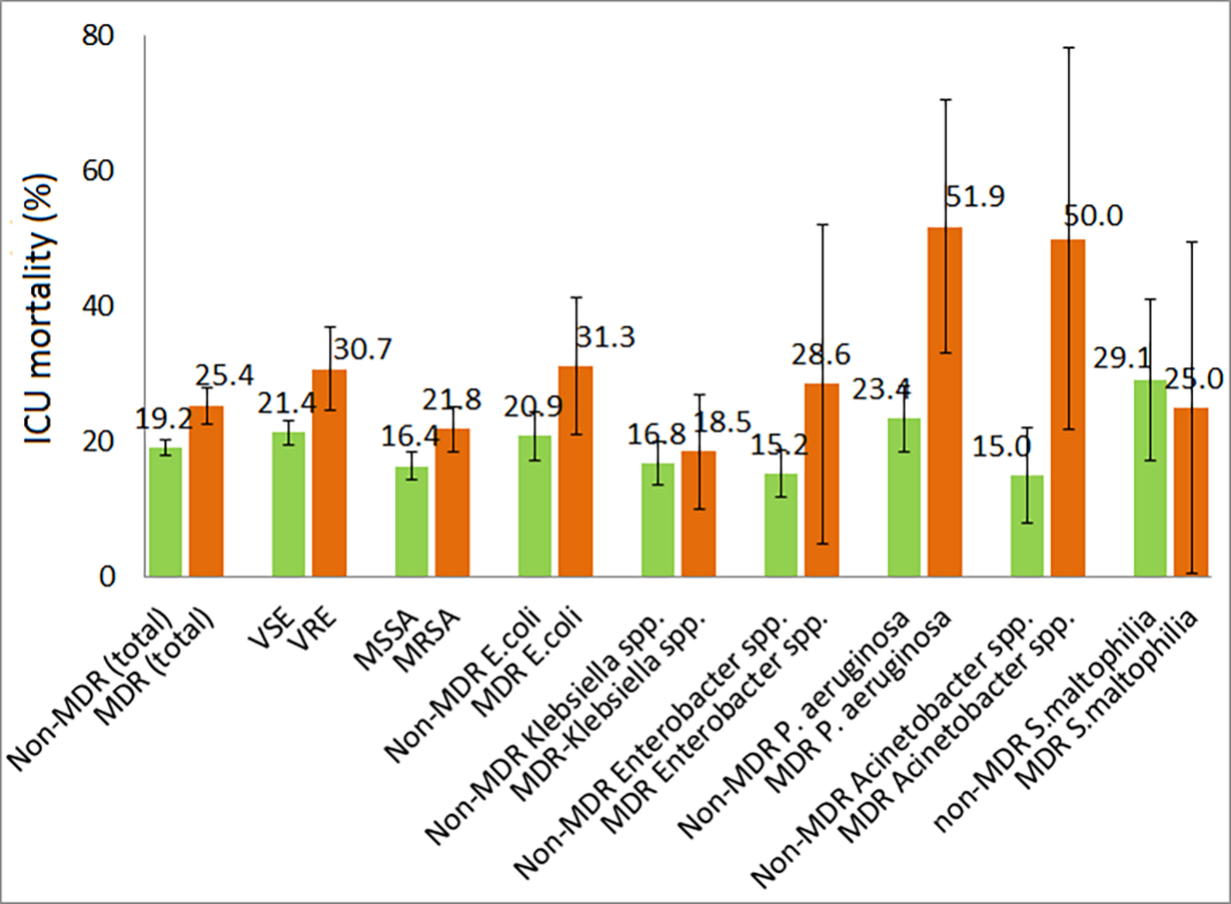
ICU mortality in patients with ICU-acquired primary bloodstream infection according to the type and resistance of pathogen. ICU, intensive care unit; MDR, Multi-drug resistance includes Methicillin resistant *S*.*aureus*, Vancomycin resistant *E*.*faecalis* and *E*.*faecium*, and multidrug resistance defined as a resistance to at least 3 antibiotic groups for *P*.*aeruginosa*, Acinetobacter *spp*., Enterobacter *spp*., *E*.*coli*, *S*.*maltophilia*, Klebsiella *spp*. and/or the resistance mechanisms ESBL for Klebsiella *spp*. and *E*.*coli*; Non-MDR, includes Vancomycin sensible Enterococcus *spp*., Methicillin sensible *S*. *aureus* and non-multi-drug resistance for *E*. *coli*, Klebsiella *spp*., Enterobacter *spp*., *P*. *aeruginosa*, Acinetobacter *spp*., *S*. *maltophilia*; Whiskers represent 95% confidence interval.

ICU mortality following mono-microbial ICU-acquired PBSI for the type of pathogen is also illustrated in Fig 1 and stratified according to the resistance of pathogen in Fig 2.

**Fig 3:**
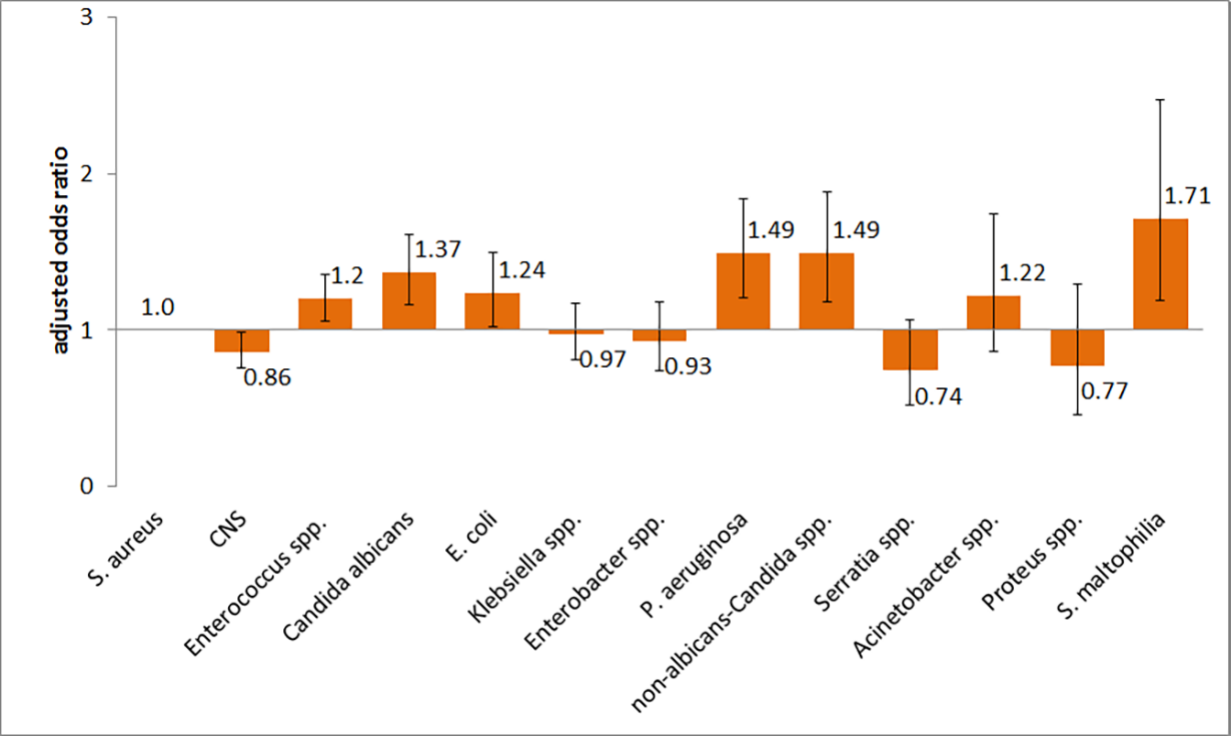
Adjusted odds ratios (AOR) for ICU mortality in patients with ICU-acquired primary bloodstream infections according to the type of pathogen. ICU, intensive care unit; CNS, coagulase negative staphylococci; Whiskers represent 95% confidence interval; S. aureus was set as reference.

Table 3 and Fig 3 show the results of the multivariable analysis for ICU mortality following PBSI of various pathogens compared to *S*.*aureus*. Adjusted by gender, age group and the type of ICU, Enterococci, *E*.*coli*, *C*.*albicans* and Non-albicans Candida *spp*., *S*. *maltophilia* and *P*.*aeruginosa* were associated with higher mortality compared to *S*.*aureus;* CNS with lower mortality respectively.

**Table 3:** Results of multivariable analysis for mortality in intensive care units (ICU) following mono-microbial primary bloodstream infection (PBSI) according to type of organism and further risk factors and confounders, ICU-KISS, 2006–2015.

Parameter	Category	AOR	95%CI	p-value
Type of organism	Coagulase negative staphylococci	0.86	0.75–0.99	0.030
	Enterococcus *spp*.	1.20	1.06–1.36	0.005
	*Candida albicans*	1.37	1.16–1.61	<.0001
	*E*. *coli*	1.24	1.02–1.49	0.029
	Klebsiella *spp*.	0.97	0.81–1.17	0.780
	Enterobacter *spp*.	0.93	0.74–1.18	0.556
	*P*. *aeruginosa*	1.49	1.21–1.84	<.0001
	*non-albicans-Candida spp*.	1.49	1.18–1.88	0.001
	Serratia spp.	0.74	0.52–1.06	0.105
	Acinetobacter *spp*.	1.22	0.86–1.74	0.262
	Proteus *spp*.	0.77	0.46–1.3	0.324
	*S*. *maltophilia*	1.71	1.19–2.47	0.004
	*S*. *aureus*	1 = reference		
Gender	Male	0.92	0.86–1	0.042
	Female	1 = reference		
Age group	≤45 years	0.46	0.4–0.52	<.0001
	46–55 years	0.70	0.62–0.78	<.0001
	56–65 years	0.79	0.72–0.86	<.0001
	>65 years	1 = reference		
Type of ICU	Medical	1.12	0.95–1.32	0.161
	Surgical	0.89	0.77–1.02	0.101
	Neurosurgical	0.44	0.27–0.72	0.001
	Cardiosurgical	1.48	1.18–1.85	0.001
	Neurological	0.52	0.39–0.71	<.0001
	Paediatric	0.55	0.38–0.8	0.002
	Other ^a^	1.02	0.8–1.3	0.887
	Interdisciplinary	1 = reference		

The multivariable analysis was performed using logistic regression analysis they were based on generalized estimating equation (GEE) models which account for this clustering effect by using an exchangeable correlation structure.

AOR, adjusted odds ratio; CI, confidence interval; ICU, intensive care unit.

^a^ other than Medical-surgical, Surgical, Medical, Cardiothoracic, Neurosurgical, Neurological or Pediatric.

Table 4 shows the results of the multivariable analysis for mortality following PBSI stratified by pathogen groups (gram-positive, gram-negative bacteria and fungi) and table 5 by multi-drug resistance groups (non-multi-drug resistant/multi-drug resistant).

**Table 4:** Results of multivariable analysis for mortality in intensive care units (ICU) following mono-microbial primary bloodstream infection (PBSI) according to group of organisms, ICU-KISS, 2006–2015.

Parameter	Category	OR	95%CI	p-value
Group of organisms	Gram-negative ^a^	1.12	1.01–1.23	0.024
	Fungi ^b^	1.42	1.25–1.62	<.0001
	Gram-positive ^c^	1 = reference		** **
Gender	Male	0.92	0.85–0.99	0.036
	Female	1 = reference		
Age group	≤45 years	0.45	0.4–0.52	<.0001
	46–55 years	0.70	0.62–0.78	<.0001
	56–65 years	0.79	0.72–0.86	<.0001
	>65 years	1 = reference		
Type of ICU	Medical	1.15	0.98–1.35	0.085
	Surgical	0.89	0.77–1.02	0.102
	Neurosurgical	0.43	0.26–0.69	0.001
	Cardiosurgical	1.45	1.16–1.83	0.001
	Neurological	0.52	0.39–0.7	<.0001
	Paediatric	0.53	0.36–0.77	0.001
	Other ^d^	1.05	0.82–1.34	0.708
	Interdisciplinary	1 = reference		

The multivariable analysis was performed using logistic regression analysis they were based on generalized estimating equation (GEE) models which account for this clustering effect by using an exchangeable correlation structure

OR, odds ratio; CI, confidence interval

Model is adjusted by age, sex and type of ICU

^a^ includes *E*. *coli*, Klebsiella *spp*., Enterobacter *spp*., *P*. *aeruginosa*, Serratia *spp*., Acinetobacter *spp*., Proteus *spp*., and *S*. *maltophilia*.

^b^ includes *C*. *albicans*, non-albicans-Candida spp.

^c^ includes coagulase negative Staphylococci, Enterococcus *spp*., *S*. *aureus;*

^d^ other than Medical-surgical, Surgical, Medical, Cardiothoracic, Neurosurgical, Neurological or Pediatric.

**Table 5:** Results of multivariable analysis for mortality in intensive care units (ICU) following mono-microbial primary bloodstream infection (PBSI) according to multidrug resistance groups, ICU-KISS, 2006–2015.

Parameter	Category	OR	95%CI	p-value
Multi-drug (MDR) resistance groups	MDR ^a^	1.36	1.21–1.52	<.0001
	Non-MDR ^b^	1 = reference		
Gender	Male	0.91	0.84–0.98	0.017
	Female	1.00	1–1	0.000
Age group	≤45 years	0.46	0.4–0.52	<.0001
	46–55 years	0.70	0.63–0.78	<.0001
	56–65 years	0.79	0.72–0.86	<.0001
	>65 years	1 = reference		
Type of ICU	Medical	1.15	0.98–1.35	0.097
	Surgical	0.92	0.8–1.06	0.236
	Neurosurgical	0.43	0.27–0.7	0.001
	Cardiosurgical	1.48	1.16–1.89	0.002
	Neurological	0.52	0.38–0.71	<.0001
	Paediatric	0.54	0.38–0.79	0.001
	Other ^c^	1.07	0.83–1.38	0.615
	Interdisciplinary	1 = reference		

The multivariable analysis was performed using logistic regression analysis they were based on generalized estimating equation (GEE) models which account for this clustering effect by using an exchangeable correlation structure

OR, odds ratio; CI, confidence interval

Model is adjusted by age, sex and type of ICU

^a^ MDR, Multi-drug resistance includes Methicillin resistant *S*.*aureus*, Vancomycin resistant *E*.*faecalis* and *E*.*faecium*, and multidrug resistance defined as a resistance to at least 3 antibiotic groups for *P*.*aeruginosa*, Acinetobacter *spp*., Enterobacter *spp*., *E*.*coli*, *S*.*maltophilia*, Klebsiella *spp*. and/or the resistance mechanisms ESBL for Klebsiella *spp*. and *E*.*coli*

^b^ Non-MDR, includes Vancomycin sensible Enterococcus *spp*., Methicillin sensible *S*. *aureus* and non-multi-drug resistance for *E*. *coli*, Klebsiella *spp*., Enterobacter *spp*., *P*. *aeruginosa*, Acinetobacter *spp*., *S*. *maltophilia*

^c^ other than Medical-surgical, Surgical, Medical, Cardiothoracic, Neurosurgical, Neurological or Pediatric.

## Discussion

The present study evaluated ICU mortality after PBSI in relation to various types of pathogen in a large cohort of more than 4.5 Mio ICU patients. PBSI with Enterococci, *E*.*coli*, *C*.*albicans* and Non-albicans Candida *spp*., *S*. *maltophilia* and *P*.*aeruginosa* were associated with higher ICU mortality compared to *S*.*aureus*, CNS were associated with significant lower ICU mortality.

The significantly higher ICU mortality rates following PBSI with *E*.*coli* and Enterococci have not been previously described and seem to be particularly interesting. Only a recent article by Ong et al. also associated enterococci PBSI with significantly higher mortality rates [7]. These authors used data from two ICUs in the Netherlands, considered ICU discharge and death as competing events. The occurrence of enterococcal bacteremia (not only PBSI) was fitted as a time time-dependent variable. The authors of this study argue that the high risk of death following entercoccal bacteremia may be a marker of impending complications that carry high risk of death in the ICU rather than being causes of death on their own. For instance, the higher mortality in Enterococci PBSI (and also *E*.*coli*) may originate in translocation from the digestive tract of patients in unstable hemodynamic conditions, such as during shock or the support of ECMO. The higher mortality of E.coli cannot be explained by the percentage of multi-drug resistant E.coli. (ICU mortality non-MDR E.coli 20.9, mortality MDR-E.coli 31.3).

Our results confirm findings from earlier studies concerning Candida *spp*. and *P*.*aeruginosa* which are known for a high mortality. In the past, Candida *spp*. was often described as leading to higher mortality. In an analysis of more than 6000 patients with BSI, Shorr et al. demonstrated that fungal organisms were associated with the highest mortality [14]. In a review, the same authors described infections caused by mixed resistant Gram-negative bacteria, extended-spectrum beta-lactamase-producing Enterobacteriaceae, multi-drug resistant *Pseudomonas aeruginosa*, and Acinetobacter species as being generally associated with increased mortality [1]. Lambert et al. described a very high mortality rate following *P*.*aeruginosa* PBSI in European ICU patients [5]. A recent cohort study from North Carolina reported that *P*.*aeruginosa* remained significantly associated with increased mortality following BSI relative to *S*.*aureus* and other gram-negative bacteria (Hazard ratio 1.43, p = 0.02) [15]. In another study from US hospitals that focused on severe sepsis, anaerobes and Methicillin-resistant *S*.*aureus* were significant predictors of mortality whereas Gram-negative bacteria had decreased mortality hazards [3].

According to a recent study with data from more than 600,000 in patients from 10 European hospitals, which focussed on all BSI due to *S*.*aureus* and Enterobacteriaceae and also considered competing risks, *S*.*aureus* had a greater impact on mortality than Enterobacteriacae. However, the authors pooled the data for all Enterobacteriacae and did not focus individual species such as *E*.*coli* [8]. In our study *E*.*coli* mortality was higher than Klebsiella *spp*., Enterobacter *spp*., Proteus *spp*. and Serratia *spp*..

Many authors have compared the mortality rates of the susceptible and resistant variants of the pathogens causing infections [5, 16–19]. We also tried to show the differences between the two groups of pathogens. But we must point out that we were not able to adjust for underlying diseases which seem to be a very important risk factor for multi-drug resistant bacteria. Only the time, from admission until onset of infection, could be considered, which may be a surrogate parameter for severity of illness. This is one of the most important limitations of our analysis. For example PBSI with *S*.*maltophilia* occur after a long stay on ICU (Median 19 days). However, the time to BSI was not significant in the multivariable analysis.

Our analysis has further limitations. First, our analysis only represents ICU mortality following ICU-acquired primary BSI. No BSI within the first three days on ICU or secondary BSI are included. Second, autopsies are very rarely performed in German ICUs, thus also precluding confirmation of a direct relationship between mortality and PBSI. Third, only mortality in the ICU was observed, but not during subsequent hospital stays. Fourth, it is impossible to provide information about the sensitivity of recording the death of the patients, but due to the use of web-based surveillance systems, the surveillance staff cannot complete an infection case without entering this information. Fifth, the analysis concentrates only on mono-microbial infections; mortality following poly-microbial infections was significantly higher. Sixthly, usually the time to event has been used to account for the risk of death, but because the time to the event “death” was not available for infected patients, such an analysis was not possible. Finally, the database does not include any information about the underlying diseases of the patients and empirical antimicrobial therapy (substances and the timing of antibiotics). Therefore, adjustment related to inappropriate initial antimicrobial therapy is impossible.

## Conclusion

In summary, out data shows substantial differences in mortality in German ICUs according to the type of pathogen. Considering the aggressive nature of the pathogens identified with significantly higher mortality rates, information on the type of pathogen is itself important for the prognosis of patients. Therefore, taking blood cultures is important and their results should be provided to clinicians as soon as possible.
